# Opportunities to reduce pollination deficits and address production shortfalls in an important insect‐pollinated crop

**DOI:** 10.1002/eap.2445

**Published:** 2021-10-19

**Authors:** Michael P. D. Garratt, G. Arjen de Groot, Matthias Albrecht, Jordi Bosch, Tom D. Breeze, Michelle T. Fountain, Alexandra M. Klein, Megan McKerchar, Mia Park, Robert J. Paxton, Simon G. Potts, Gesine Pufal, Romina Rader, Deepa Senapathi, Georg K. S. Andersson, Olivia M. Bernauer, Eleanor J. Blitzer, Virginie Boreux, Alistair J. Campbell, Claire Carvell, Rita Földesi, Daniel García, Lucas A. Garibaldi, Peter A. Hambäck, Giorgi Kirkitadze, Anikó Kovács‐Hostyánszki, Kyle T. Martins, Marcos Miñarro, Rory O’Connor, Rita Radzeviciute, Laura Roquer‐Beni, Ulrika Samnegård, Lorraine Scott, Nicolas J. Vereecken, Felix Wäckers, Sean M. Webber, George Japoshvili, Aigul Zhusupbaeva

**Affiliations:** ^1^ Centre for Agri‐Environmental Research, SAPD University of Reading Reading RG6 6AR United Kingdom; ^2^ Wageningen Environmental Research (WENR) P.O. Box 47 6700 AA Wageningen The Netherlands; ^3^ Eidgenössisches Departement für Wirtschaft Agroscope Reckenholzstrasse 191 CH‐8046 Zürich Switzerland; ^4^ CREAF Universitat Autònoma de Barcelona Cerdanyola del Vallès 08193 Catalunya Spain; ^5^ NIAB EMR East Malling Kent ME19 6BJ United Kingdom; ^6^ Chair of Nature Conservation and Landscape Ecology Albert‐Ludwigs‐University 79106 Freiburg Germany; ^7^ Geography, Archaeology and the Environment University of Worcester Worcester WR2 6AJ United Kingdom; ^8^ Department of Biological Sciences North Dakota State University Fargo North Dakota 58201 USA; ^9^ Institute for Biology Martin Luther‐University Halle‐Wittenberg Hoher Weg 8 Halle (Saale) 06120 Germany; ^10^ School of Environment and Rural Science University of New England Armidale New South Wales 2351 Australia; ^11^ Department of Biology Lund University 223 62 Lund Sweden; ^12^ Hawkesbury Institute for the Environment Western Sydney University Richmond 2753 New South Wales Australia; ^13^ Department of Biology Carroll College Helena 59601 Montana USA; ^14^ Embrapa Amazônia Oriental 66095‐903 Belém Brazil; ^15^ UK Centre for Ecology & Hydrology OX10 8BB Wallingford United Kingdom; ^16^ Lendület Ecosystem Services Research Group, Institute of Ecology and Botany, Centre for Ecological Research 2163 Vácrátót Hungary; ^17^ Depto. Biología de Organismos y Sistemas (Universidad de Oviedo) and Instituto Mixto de Investigación en Biodiversidad (IMIB, CSIC‐Universidad de Oviedo‐Principado de Asturias) C/Catedrático Rodrigo Uría s/n Oviedo E‐33006 Asturias Spain; ^18^ Universidad Nacional de Río Negro, Instituto de Investigaciones en Recursos Naturales, Agroecología y Desarrollo Rural San Carlos de Bariloche Río Negro Argentina; ^19^ Consejo Nacional de Investigaciones Científicas y Técnicas Instituto de Investigaciones en Recursos Naturales, Agroecología y Desarrollo Rural San Carlos de Bariloche Río Negro Argentina; ^20^ Department of Ecology, Environment and Plant Sciences Stockholm University 106 91 Stockholm Sweden; ^21^ Institute of Entomology Agricultural University of Georgia 0159 Tbilisi Georgia; ^22^ Department of Biology McGill University Montréal H3A 0G4 Québec Canada; ^23^ Servicio Regional de Investigación y Desarrollo Agroalimentario (SERIDA) Apdo. 13 Villaviciosa E‐33300 Asturias Spain; ^24^ Molecular Evolution and Animal Systematics Institute of Biology University of Leipzig Talstraβe 33 04103 Leipzig Germany; ^25^ Department of Biology Lund University 223 62 Lund Sweden; ^26^ School of Biological Sciences Queen’s University Belfast BT9 7BL Belfast United Kingdom; ^27^ Agroecology Lab Université libre de Bruxelles (ULB) Boulevard du Triomphe CP 264/2 B‐1050 Brussels Belgium; ^28^ Lancaster Environment Centre Lancaster University LA1 4YQ Lancaster United Kingdom; ^29^ Academy of Public Administration under the President of the Kyrgyz Republic 237 Panfilova str. Bishkek Kyrgyzstan

**Keywords:** agro‐ecology, apples, *Malus domestica*, pollinators, sustainable crop production

## Abstract

Pollinators face multiple pressures and there is evidence of populations in decline. As demand for insect‐pollinated crops increases, crop production is threatened by shortfalls in pollination services. Understanding the extent of current yield deficits due to pollination and identifying opportunities to protect or improve crop yield and quality through pollination management is therefore of international importance. To explore the extent of “pollination deficits,” where maximum yield is not being achieved due to insufficient pollination, we used an extensive dataset on a globally important crop, apples. We quantified how these deficits vary between orchards and countries and we compared “pollinator dependence” across different apple varieties. We found evidence of pollination deficits and, in some cases, risks of overpollination were even apparent for which fruit quality could be reduced by too much pollination. In almost all regions studied we found some orchards performing significantly better than others in terms of avoiding a pollination deficit and crop yield shortfalls due to suboptimal pollination. This represents an opportunity to improve production through better pollinator and crop management. Our findings also demonstrated that pollinator dependence varies considerably between apple varieties in terms of fruit number and fruit quality. We propose that assessments of pollination service and deficits in crops can be used to quantify supply and demand for pollinators and help to target local management to address deficits although crop variety has a strong influence on the role of pollinators.

## Introduction

Demand for crops that rely on insect pollinators is increasing on a global scale (Aizen et al. 2019). Yet, due to multiple threats (Vanbergen and Initiative 2013, Potts et al. 2016), populations of both wild and managed pollinators may not meet present or future demands for pollination service provision, compromising production by limiting yield and quality of crops. We are increasingly aware of the significant contribution that pollinators make to global food production, particularly of nutritionally important crops (Smith et al. 2015). In addition, as evidence of yield deficits emerge (Garibaldi et al. 2016), there is a need to ensure pollination services are supported through policy and practice (Dicks et al. 2016, Potts et al. 2016, Garibaldi et al. 2019). Avoiding mismatches between the supply of, and demand for, this valuable ecosystem service is vital for future sustainable food production.

Cost‐effective management of insect pollination services by farmers, land managers, and policy makers requires coordinated action at field, farm and landscape scales (Garibaldi et al. 2019), and both wild and managed pollinators may be required to ensure adequate pollen transfer and optimal crop production (Garibaldi et al. 2014, Isaacs et al. 2017). However, matching pollination supply and demand to optimize yield and quality is not always straightforward as it requires combined knowledge of both a crop’s breeding system (Hudewenz et al. 2013, Benjamin and Winfree 2014, Garratt et al. 2016), as well as the influence of environmental and management context on pollination. For example, agronomic inputs including fertilizers and irrigation (Klein et al. 2015, Garratt et al. 2018), biological factors such as pest pressure (Barber et al. 2012, Bartomeus et al. 2015, Sutter and Albrecht 2016, Samnegård et al. 2019), and even environmental and climatic variables (Bishop et al. 2016), can result in complex interactions that affect the contribution of pollinators to crop yield (Tamburini et al. 2019).

Apples are a globally significant crop valued at US$45 bn annually (FAOStat 2018), with high economic and nutritional value. They are grown by large‐scale commercial operations and small‐scale farmers alike. Apple production relies on insect pollination (Ramírez and Davenport 2013, Cross et al. 2015, Demestihas et al. 2017), but the degree of pollination by either managed or wild pollinators varies (Stern et al. 2001, Martins et al. 2015, Földesi et al. 2016, Joshi et al. 2016, Geslin et al. 2017), and the delivery of pollination service has been found to depend on apple variety (Garratt et al. 2016). Despite relatively few reported examples (Garratt et al. 2014, Blitzer et al. 2016), pollination deficits could arise due to pollinator loss, poor weather during flowering, insufficient availability of compatible pollen, or a number of other factors. Yet we are not sure in which regions and varieties this is indeed a potential hazard, or if in fact deficits already exist.

Sustainable crop production depends on approaches that help to predict potential and actual risks of yield losses arising from pollination shortfalls and identifying orchards where production is limited to target interventions. Using a global dataset, we set out to answer the following research questions: (1) How widespread are pollination deficits in apples and to what extent do these vary among orchards and countries? (2) How does crop variety influence dependence on pollinators and pollination deficits? and (3) How does pollination effect aspects of both fruit yield and fruit quality across different apple varieties?

## Methods

### Datasets

We gathered datasets on insect pollination in apples from regions around the world, including intensive commercial orchards and low‐intensity smaller‐scale production. The analysis involved working with raw datasets and data holders were identified and approached following a workshop held on apple pollination as part of the “Sustainable Pollination in Europe” Super‐B COST Action Project to which European and other international researchers were invited. Studies were included if they involved manipulation of apple blossoms. Manipulations included pollinator exclusion using net bags, supplementary pollination, whereby pollen was applied by hand using compatible pollen from local pollinizer trees or neighboring varieties, and open “controls” accessible to insect visits. Studies recorded metrics of apple pollination, including early fruit set and seed number per apple, or apple production such as fruit set at harvest and fruit quality in terms of apple size (maximum width mm), weight (g), firmness (kg cm^–1^ measured using a penetrometer) and sugar content (% brix measured using a refractometer). The analyses in which each study was involved depended on data availability and metrics taken, so not all studies were incorporated into all analyses. In total, data from 14 countries and five continents was analyzed, comprising 36 apple varieties across 356 orchards (Appendix S1: Table S1).

### Calculating pollinator dependence, service and deficits

**Fig. 1:**
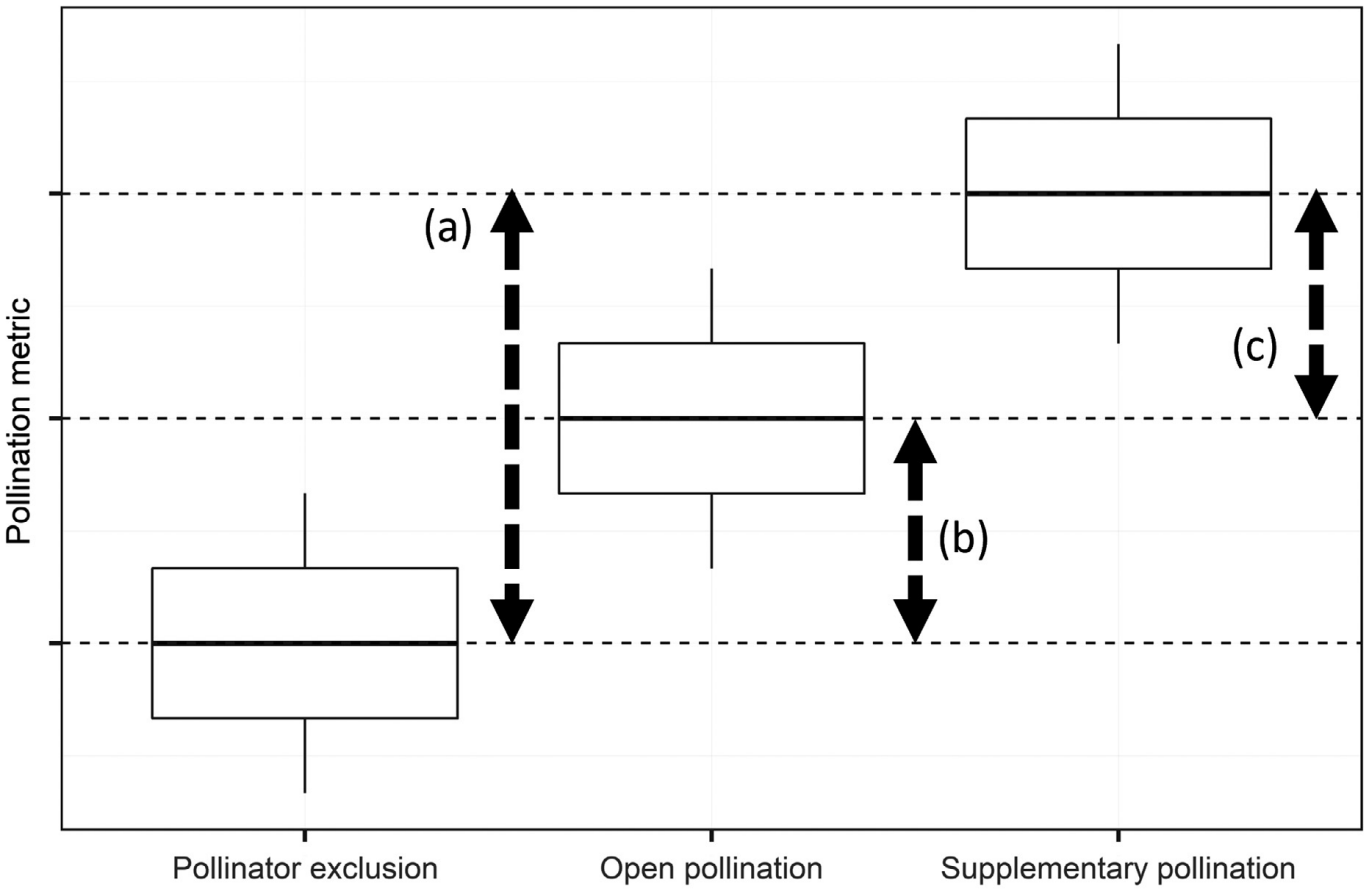
Theoretical output achieved under different experimental treatments. (a) Pollinator dependence, i.e., the level to which insects could contribute to pollination. (b) Pollination service, i.e., the extent to which pollinator dependence is met by ambient pollination conditions. (c) Pollination deficits in apple pollination or production, i.e., the shortfall of ambient pollination below maximum potential pollination.

Using data from pollinator exclusion, open pollination and supplementary hand pollination (from this point forwards supplementary pollination) treatments, levels of pollinator dependence, pollination service, and pollination deficit were assessed across orchards, countries, and apple varieties for a number of apple response metrics. These response metrics can be divided into two broad categories: “pollination” and “production.” We used early fruit and seed number to represent “pollination” as they reflected the level of compatible pollen delivery to apple flowers but are not intrinsically of value to farmers. Final fruit set at harvest, yield (fruit set × fruit weight), and apple quality (size, sugar content, firmness) represent final crop outputs for farmers and were considered as “production” metrics. “Pollinator dependence” represents the potential contribution of insect pollinators to these metrics, and was calculated by subtraction of the output achieved following the exclusion of insect pollinators, from the maximum achievable by supplementary pollination. “Pollination service” represents the realized contribution of insects to pollination at any given place and time. It was calculated by subtracting the output from pollinator exclusion treatments from that recorded under open pollination treatments. Finally, “Pollination deficit” represent a shortfall in output due to a lack of pollination and was calculated by subtracting outputs from open pollination treatments from those achieved under supplementary pollination (Fig. 1).

### Pollination service and deficits across countries, orchards and varieties

To assess the extent of yield loss in orchards resulting from insufficient pollination (a pollination deficit), datasets from studies that had implemented supplementary pollination and open pollination treatments in at least three orchards of the same variety in the same country and included production variables, namely final fruit set and fruit weight, were analysed. This included data for 11 apple varieties across five countries. Pollination service and deficit were calculated as a proportion of maximum yield achieved in either open or supplementary treatments, whichever was greatest. To compare between countries and varieties, the pollination deficit (± 95% confidence limits) was calculated across orchards for each country and variety combination. Countries and varieties for which confidence limits fell outside a zero deficit were considered to have a significant system‐level deficit for yield.

To identify orchards with a significant pollination deficit relative to other orchards in that country growing the same apple variety, data were used from orchards where supplementary and open pollination treatments were implemented on at least three replicate locations within the orchard. Mean pollination deficits were then calculated for each orchard. If the 95% confidence limits for each orchard did not include the mean of the orchard with the pollination deficit closest to zero within that variety and country, the orchard was considered as having a significant yield deficit requiring pollination management. Due to the effects of experimental scale on assessments of pollination (Bishop et al. 2020, Webber et al. 2020), only orchards within each country and variety where experimental manipulations used the same unit of assessment (e.g. tree branch) were compared. To assess the relationship between the extent of pollination deficits and the level of pollination service measured in each orchard, a linear mixed effect model was used, with orchard, apple variety, study, and country as nested random effects.

### Differences in pollination dependence between varieties

Linear mixed effects models were used to compare pollinator dependence of both pollination and production metrics between apple varieties. In total, 17 studies involving 26 apple varieties included a supplementary pollination treatment and pollinator exclusion treatment and recorded at least one pollination or production metric. Pollination treatment (pollinator exclusion or supplementary pollination), variety, and their interaction were included as fixed effects in the model. Study, orchard, and sampling location within orchards were included as nested random effects. To test for a significant interaction between pollination treatment and apple variety (*P* > 0.05) models with and without the interaction term were compared using a maximum likelihood ratio test. Both early and final percent fruit set were arcsine transformed, and seed number and firmness log‐transformed prior to analysis. Model residuals were checked to ensure that they met model assumptions. To assess for significant treatment effects on pollination and production metrics for each variety, post‐hoc Tukey tests were carried out.

### Relationships between pollination and production

To examine the relationships between pollination and production, the relationships between seed number and final fruit set or apple size were investigated using linear mixed effects models. Variety and either seed number or percentage fruit set and their interaction were included in the models as fixed effects. Study, orchard, and sampling location within orchard were treated as random effects. Again, seed number was log‐transformed prior to analysis. All statistical analyses were carried out in R v. 4.0.3 using packages *lme4* (Bates et al. 2014), *nlme* (Pinheiro et al. 2013), and *multcomp* (Hothorn et al. 2008, R_Core_Team 2017).

## Results

### Pollination service and deficits across countries, orchards and varieties

**Fig. 2:**
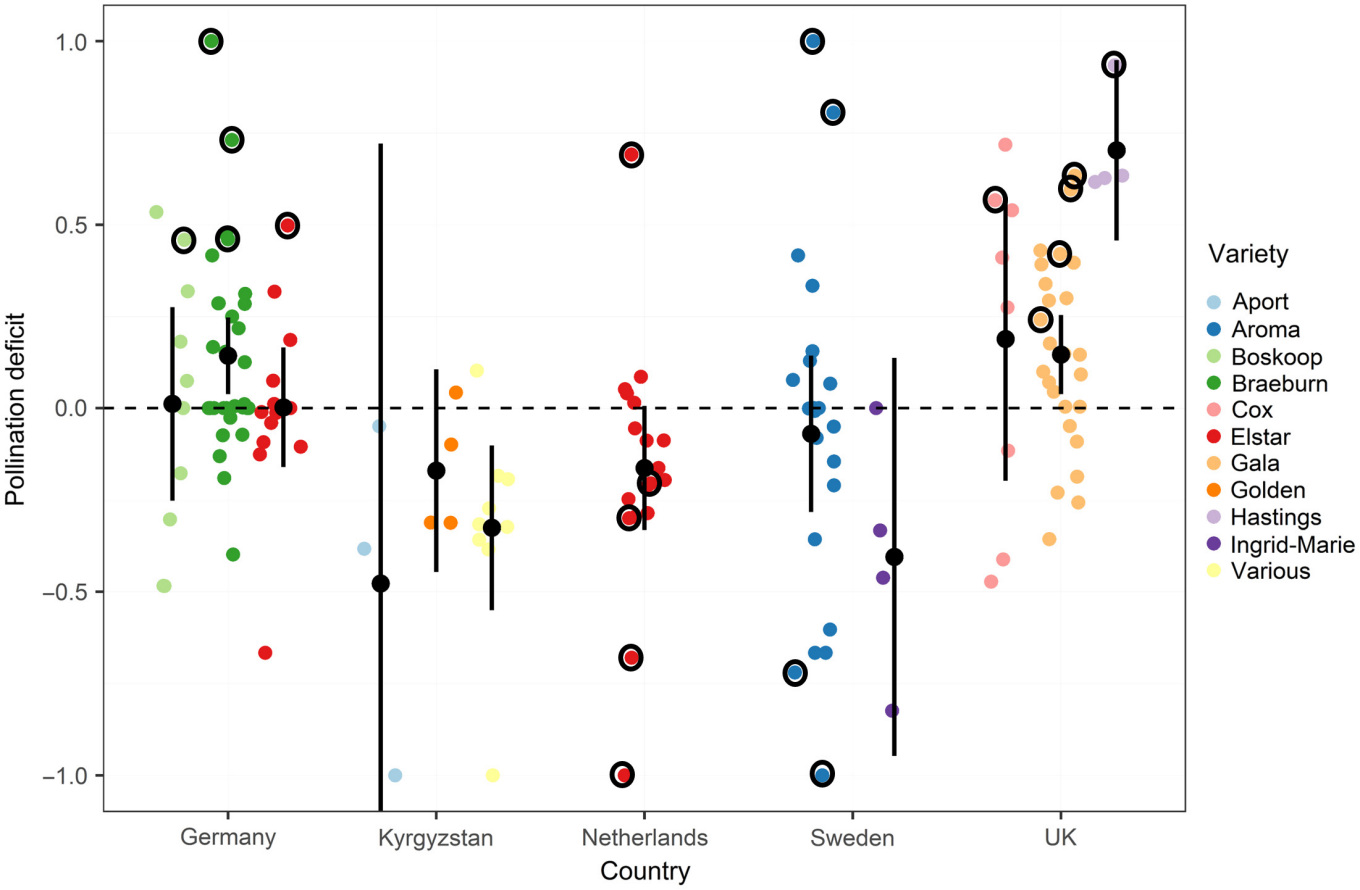
Deficits in yield due to suboptimal pollination in orchards (individual points) separated by apple variety (color) per country. Mean and 95% CI are shown for each variety within each country (“various” refers to orchards made up of multiple varieties). A positive deficit occurs when yield is greater under supplementary pollination compared with open pollination treatments, and a negative deficit occurs when yield in open treatments is greater than for supplementary pollination. Points in circles represent individual orchards with a significant deficit in yield relative to the best performing orchard growing that variety in that country (i.e., the orchard with a pollination deficit closest to 0).

Data from 11 varieties and five countries included open, pollinator exclusion and supplementary pollination treatments and measured final fruit set and apple weight, allowing for orchard‐level assessments of pollination service and pollination deficits for yield. Orchards growing three apple varieties from two countries showed a significant pollination deficit overall: Gala and Hastings orchards in the UK; as well as Braeburn orchards in Germany (Fig. 2). Orchards growing mixed varieties of apples in Kyrgyzstan had a significantly negative deficit, indicating that supplementary pollination reduced the yield compared with open pollination. At least one orchard per country and apple variety showed significant pollination deficits relative to the best performing orchard in that country growing the same variety (Fig. 2), except for in Kyrgyzstan where multiple pollination assessments per orchard were not made, so individual orchard comparisons were not possible.

**Fig. 3:**
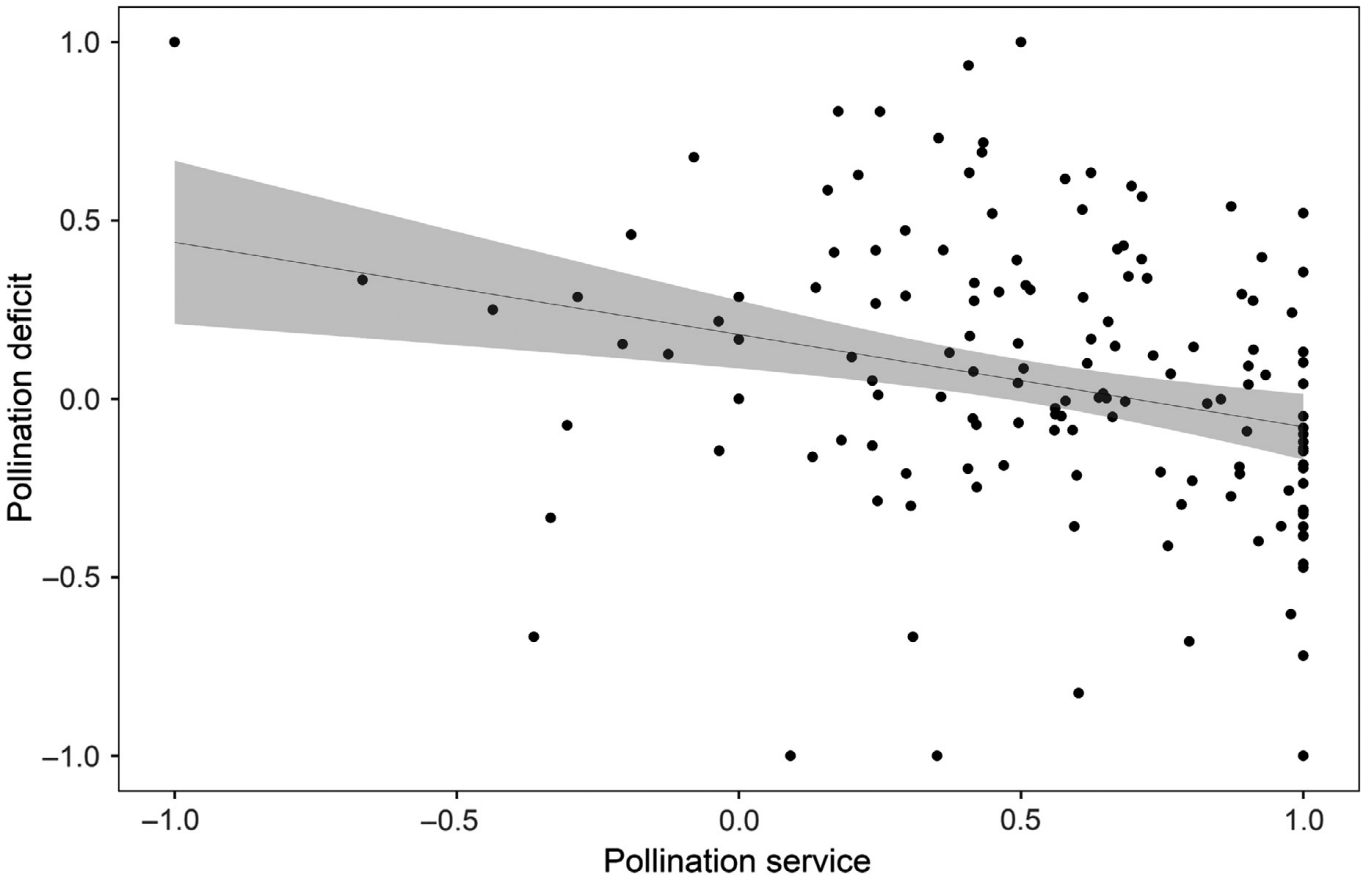
Relationship between pollination service (i.e., the current contribution of insects to yield) and pollination deficits (a shortfall in yield due to suboptimal pollination) for apple orchards across countries and varieties. Linear model and 95% confidence limits are shown.

A negative linear relationship between pollination deficits and pollination service for yield was observed (*t* = −3.40, *P* < 0.001) (Appendix S1: Table S2), indicating that orchards with high values of pollination service were less likely to have pollination deficits (Fig. 3).

### Differences in pollinator dependence between varieties

**Fig. 4:**
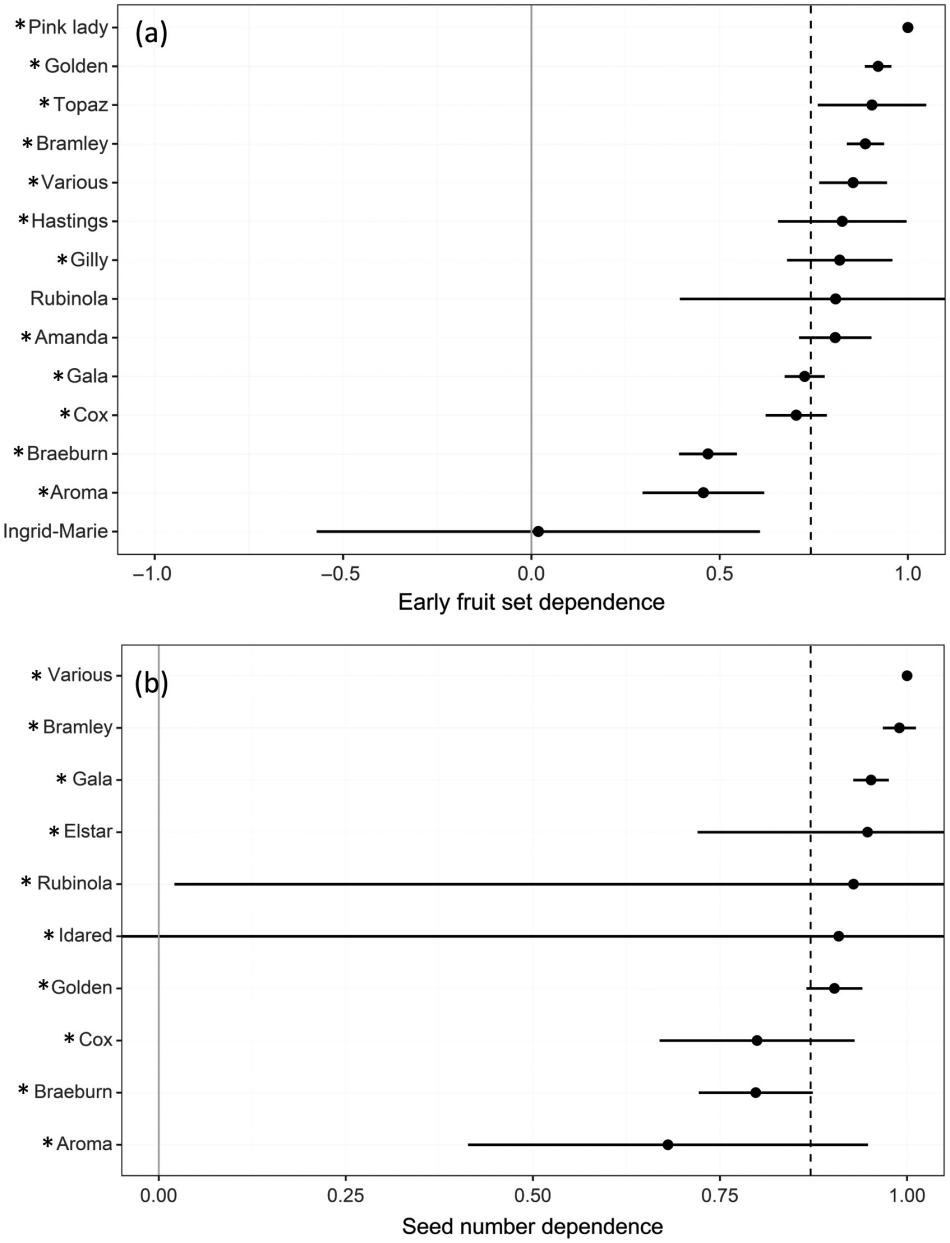
The extent to which early fruit set (a) and seed number (b) of different apple varieties depend on pollination using available data from all orchards and countries. Mean pollinator dependence and 95% CI are shown for each variety and grand mean across varieties shown as a dashed line. Varieties marked with “*” indicate those with significant differences found between supplementary pollination and pollinator exclusion treatments (*P* < 0.05).

The pollinator dependence of apples varied considerably among varieties for metrics of pollination, with mean dependence ranging from 0.0 to 1.0 for early fruit set and 0.68 to 1.0 for seed number (Fig. 4). There was a significant interaction between variety and pollination treatment for both early fruit set (*F* = 18.79, *P* < 0.001) and seed number (*F* = 6.20, *P* < 0.001). A significant effect of pollination treatment was observed for 12 out of 14 varieties for early fruit set and all 10 varieties for seed number (Fig. 4a, b; Appendix S1: Tables S3, S4).

**Fig. 5:**
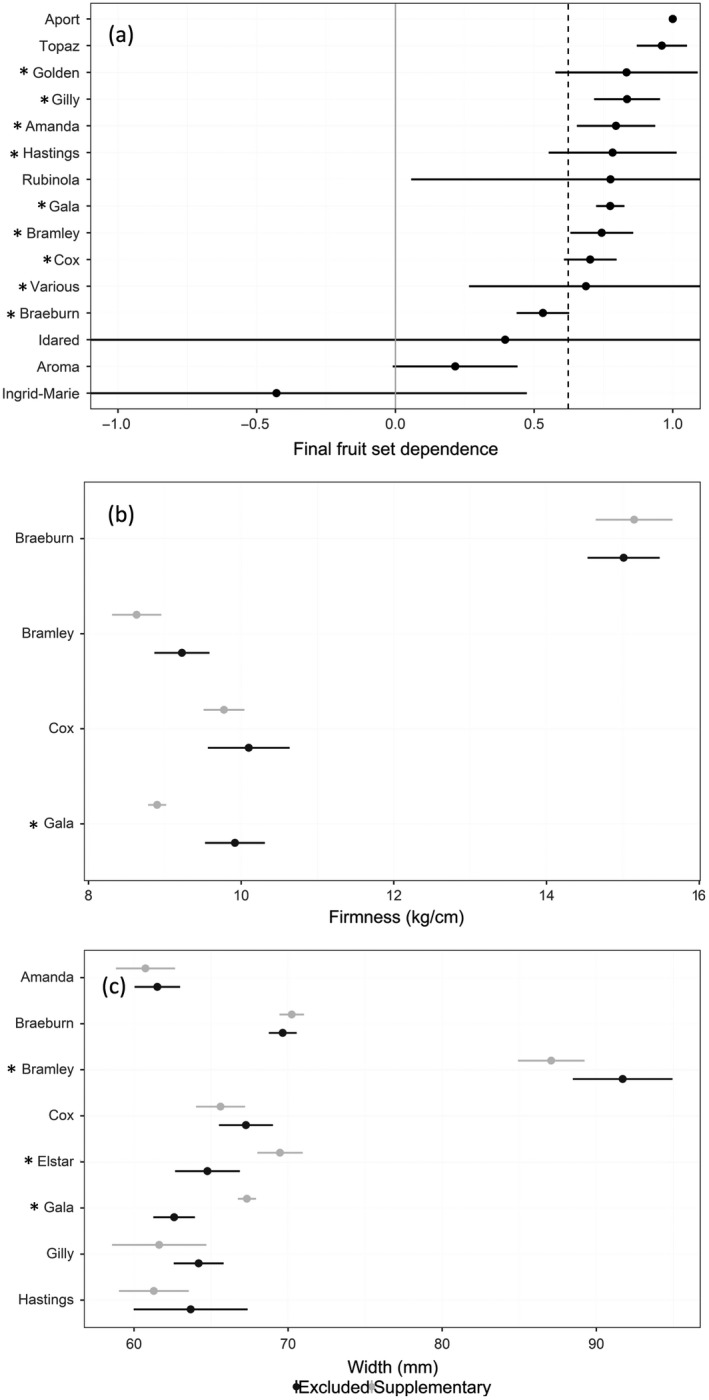
The extent to which the production of different apple varieties, measured as final fruit set (a), and fruit quality, in terms of firmness (b) and size (c), depend on pollination using available data from all orchards and countries. Mean pollinator dependence and 95% CI are shown for each variety and grand mean for fruit set across varieties is shown as a dashed line in (a). Varieties marked with “*” indicate significant differences between pollinator exclusion and supplementary pollination treatments (*P* < 0.05).

The pollinator dependence of apple production in terms of final fruit set and quality also varied considerably among varieties (Fig. 5). Mean dependence of final fruit set ranged from −0.42 and 1.0 depending on variety, with a significant interaction between experimental treatment and variety (*F* = 8.61, *P* = <0.001) and significant differences between pollination treatments were observed for 9 out of 15 varieties (Appendix S1: Table S5). There was also an interactive effect of variety and pollination treatment on apple size (*F* = 8.20, *P* < 0.001), and firmness (*F* = 3.64, *P* = 0.012) (Appendix S1: Tables S6, S7). In contrast, interactive effects of variety and pollination treatment were not found for sugar content (*F* = 0.98, *P* = 0.42). When all varieties were considered together there was a significant difference in sugar content observed between pollination treatments (*F* = 7.19, *P* = 0.006) but not between apple varieties (*F* = 1.97, *P* = 0.09) (Appendix S1: Table S8).

### Relationship between pollination and production

**Fig. 6:**
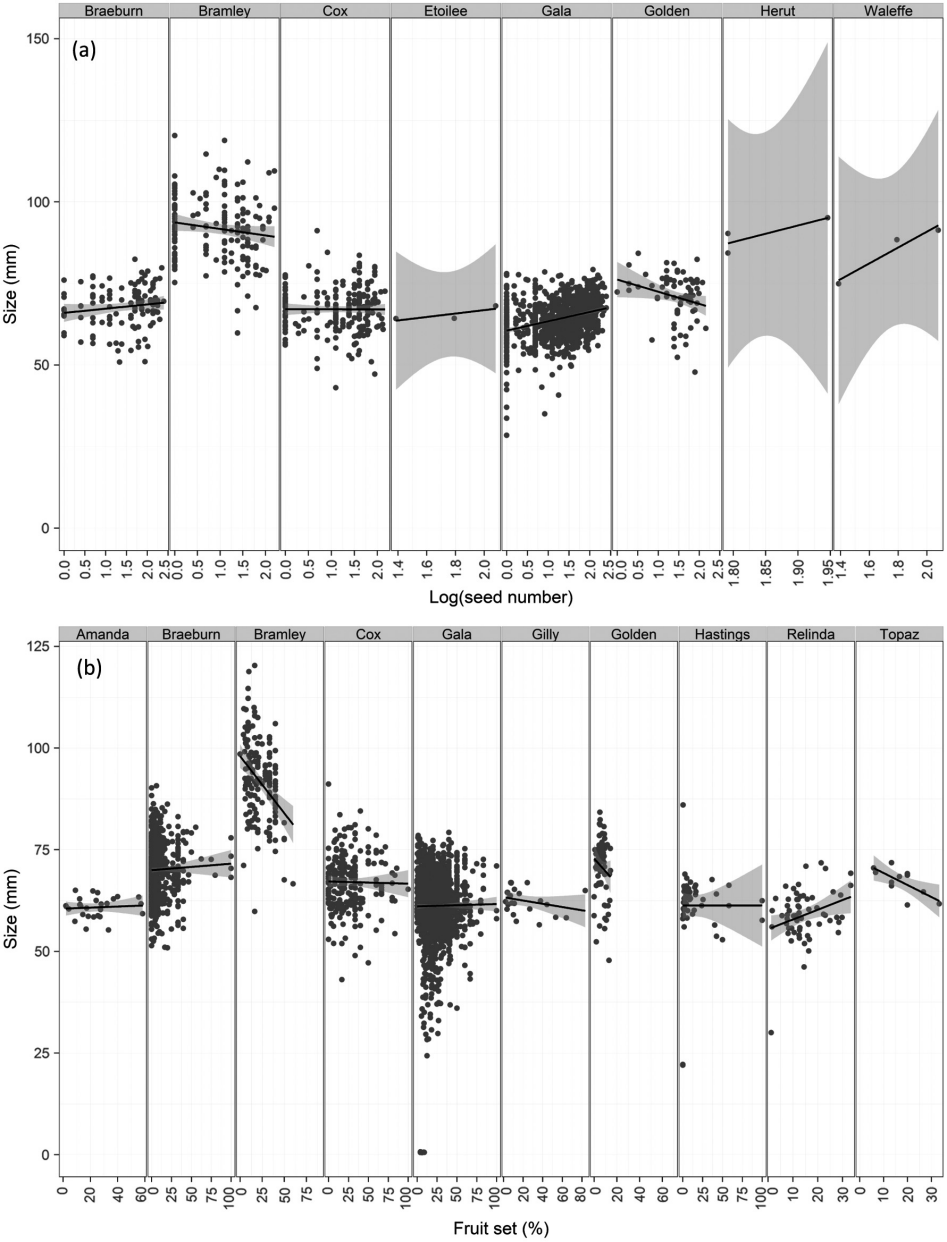
Relationship between metrics of pollination and production in different apple varieties including (a) seed number and apple size at harvest, and (b) final fruit set and apple size at harvest for multiple apple varieties. Only varieties with at least three data points were included. Linear model and 95% confidence limits are shown.

Metrics of pollination and production were interrelated, but the direction of these relationships varied among varieties. The relationship between seed number and fruit size depended on apple variety (*F* = 5.83, *P* < 0.001) (Appendix S1: Table S9). Seven varieties showed a positive relationship, in which apples containing more seeds were also larger, while two varieties showed a negative relationship. The relationship between final fruit set and fruit size was also variety dependent (*F* = 3.45, *P* < 0.001) (Appendix S1: Table S10); some varieties exhibited a positive, some a negative, and others no relationship (Fig. 6).

## Discussion

Individual orchards and regions experiencing pollination deficits (i.e. production shortfalls due to pollination) were identified in this study (Fig. 2) and pointed to an opportunity for optimizing pollination management. Observed deficits could be the result of numerous factors including insufficient abundance and diversity of wild pollinators (Martins et al. 2015, Blitzer et al. 2016, Grab et al. 2019), a lack of availability or awareness of the need for managed pollinators (Stern et al. 2001, Geslin et al. 2017), suboptimal fruit management practices such as thinning (Link 2000) or the lack of appropriate “pollinizer” trees providing compatible pollen (Ramírez and Davenport 2013), agrochemicals impacts (Stanley et al. 2015), or even overpollination (Sáez et al. 2014). In most study countries, we observed at least one orchard with optimal pollination services (i.e., deficits close to 0), which indicated that there were no regional constraints on achieving optimal pollination. These orchards with no or lower deficits could act as “agroecological lighthouse” orchards (Nicholls and Altieri 2018) providing a management and contextual role model for others to follow and help to identify factors that limit production on farms with deficits, or to provide a platform to share management practices that ensure optimal pollination. This would allow for directed management toward achieving better pollination services. Best practices would need to be shared using effective tools and techniques, and exploit appropriate networks for each region and group of growers (Ingram 2008, Klerkx and Jansen 2010).

The link between pollination deficits in yield and level of pollination services across orchards demonstrated in this study indicates that an important driver of production deficits is low levels of insect pollination. These yield deficits could be addressed through habitat management (Blaauw and Isaacs 2014, Földesi et al. 2016, Sutter et al. 2018), by avoiding pesticides harmful to wild pollinators (Park et al. 2015, Stanley et al. 2015) or through the effective use of managed pollinators (Stern et al. 2001, Geslin et al. 2017). In the past, the uptake of practices to promote biodiversity‐based ecosystem services has been slow, however, identifying deficits in production metrics such as yield and quality, familiar to farmers, may encourage uptake of ecologically responsible practices (Kleijn et al. 2019). To increase the likelihood of positive action taking place, farmers and their advisers can be encouraged to employ methods similar to those used in this study to assess their own levels of pollination service and deficit (i.e. by bagging flowers and carrying out supplementary pollination), therefore becoming more engaged with the process and gathering targeted data on which they can make informed management decisions (Garratt et al. 2019). The scale at which supplementary and pollinator exclusion techniques are used, and whether manipulations are carried out on the whole tree, single branches, or groups of flowers can influence the resulting deficits (Bishop et al. 2020, Webber et al. 2020), therefore widespread assessment should use common protocols and focus on collecting production metrics relevant to growers, such as yield (Garratt et al. 2019).

Our study has identified yield deficits due to suboptimal pollination in apple production and the extent to which these vary across orchards. Although we showed that these deficits are likely to be a result of insufficient pollination by insects, additional research is required to identify exact causes. If, for example, there is a landscape‐wide limitation in wild pollinator abundance (Martins et al. 2015, Park et al. 2015, Kremen and Merenlender 2018, Winfree et al. 2018), then the capacity of individual farmers to control this is limited. In such circumstances, amendments to policy may be necessary to promote large‐scale collaborative action (Garibaldi et al. 2019). This is particularly relevant to regions in the UK and Germany and for the varieties Hastings and Braeburn, respectively, as overall these orchards appear to be experiencing a deficit, reflective of a regional or varietal, rather than orchard‐scale challenge. That apples are effectively pollinated by a wide variety of insects (Pardo and Borges 2020), even away from their native range, means that management targeting different and locally available pollinators could deliver benefits.

Similarly to other insect‐pollinated crops (Hudewenz et al. 2013, Benjamin and Winfree 2014) we observed that dependence on insect pollination varied considerably between apple varieties in both pollination, with seed number dependence ranging from 0.68 to 1.0, and production, with dependence of fruit set at harvest between −0.42 and 1.0. This negative dependence could indicate that some varieties are potentially at risk of overpollination, although this negative dependence was not significant for any variety. It should also be noted that the response of a tree to supplementary pollination or pollinator exclusion may be influenced by external factors such as orchard management practice or seasonal conditions during the study year and could affect the level of dependence measured. Without measuring the dependence of different varieties across multiple regions and years, it is not possible to account fully for these confounding effects. However, the extent of variation in pollinator dependence that we present in this study demonstrate that variety is a key factor to consider when implementing pollinator management strategies in apple orchards. The level of dependence on insect pollinators will ultimately dictate the vulnerability of production to pollinator declines, or the extent of opportunities available to increase production. We found examples in which varieties were entirely pollinator dependent for fruit and seed number, while a minority appeared relatively self‐compatible (e.g. Ingrid‐Marie) due to unknown factors (e.g., parthenocarpy, floral anatomy promoting self‐pollination). Breeding self‐compatibility into crops has been proposed as a possible strategy to reduce their vulnerability to limited pollination provided by insects (Knapp et al. 2017). Such an approach could be adopted for apples, targeting at‐risk regions or varieties. However, self‐pollination can potentially have an impact on the micronutritional and other quality parameters of fruit (Eilers et al. 2011, Klatt et al. 2014). Furthermore, self‐incompatibility is the norm in commercial apple varieties (Matsumoto 2014) and, as apples are a long‐lived perennial crop, breeding takes decades. Also, perhaps more than any other crop, the apple variety is a key component of consumer preferences, so the continued demand for many current popular apple varieties that are self‐incompatible is likely.

Overpollination is a risk in some crops (Sáez et al. 2014), and we found evidence of overpollination in apples, with some individual orchards demonstrating significantly negative pollination deficits, indicating that enhancing pollination compared with current levels could harm production. Across our studies, compatible pollen was used and care was taken not to damage flowers when implementing supplementary pollination treatments, but ineffective manual pollination, poor pollen quality, or stigmas clogged with incompatible pollen can lead to underestimates of deficits; if assessment of pollination services is to become widespread then methods should be standardized (Webber et al. 2020). However, our results identified a mechanism for this apparent overpollination in apples, as some varieties showed that increasing fruit set or seed number, metrics particularly responsive to insect pollination (Garratt et al. 2014, 2016), resulted in reduced fruit quality in terms of size. This was particularly prominent for Bramley, Topaz and Golden Delicious. This overpollination is likely to be a result of resource limitation in trees; when fruit set is high, the maximum fruit size achieved by the tree is reduced. This is an example of a trade‐off between pollination and other inputs (Garratt et al. 2018, Tamburini et al. 2019). In apples, growers are aware of this trade‐off and use mechanical and chemical flower and fruit thinning practices to optimize fruit number and, therefore, fruit quality which underpins the economic output in many regions (Link 2000, Garratt et al. 2014). For other varieties, increasing seed number through better insect pollination increased apple size (e.g. Gala, Braeburn).

Optimizing pollination services through abundant and diverse pollinator communities is likely to ensure resilience in pollination services (Bartomeus et al. 2013, Brittain et al. 2013) and sufficient fruit set every year, provided that thinning and pruning practices are effective in years with high fruit load. Our results highlight an opportunity for farmers to accrue benefits by monitoring pollination services and crop production on their farms (Garratt et al. 2019) and by using appropriate management practices in those apple varieties and individual farms to limit pollination deficits and overpollination. Furthermore, consistent multiyear assessments of insect‐pollinated crops would expand our understanding of crop pollination and the limits to yield across the globe. Implementing standardized methods across more sites, more varieties, and more years would provide important insight into the changing status of pollination services across space and time (Breeze et al. 2021).

## Conclusions

In this study, adopting apple as an example of an important insect‐pollinated crop, we showed how the assessment of pollination services could be used to quantify and compare pollination deficits across orchards. Such approaches could be applied to other insect‐pollinated crops to understand the extent of pollination service limitations on production. Moreover, orchardists can follow the example of fields, farms, and regions where pollination is optimal, taking them as model systems to help develop management approaches that would improve pollination services. Such approaches to matching pollination supply and demand are most effective when farmers are able to assess their own crop pollination status, allowing them to make management decisions on field‐by‐field and season‐to‐season bases. Supplementary and pollinator exclusion techniques can be adapted and made user friendly, allowing farmers to adapt these techniques for their own crops (Garratt et al. 2019). Ultimately if we are to understand and mitigate the consequence of pollinator declines globally, then we need to make assessments and take action locally; the approaches identified in this study are a step toward this.

## Supporting information

Appendix S1

## Data Availability

Data sets (Garratt et al. 2021) analyzed in this current study are available through the University of Reading Data Archive at: http://dx.doi.org/10.17864/1947.314.

## References

[eap2445-bib-0001] Aizen, M. A. , et al. 2019. Global agricultural productivity is threatened by increasing pollinator dependence without a parallel increase in crop diversification. Global Change Biology 25:3516–3527. 31293015 10.1111/gcb.14736 PMC6852307

[eap2445-bib-0002] Barber, N. A. , L. S. Adler , N. Theis , R. V. Hazzard , and E. T. Kiers . 2012. Herbivory reduces plant interactions with above‐ and belowground antagonists and mutualists. Ecology 93:1560–1570. 22919903 10.1890/11-1691.1

[eap2445-bib-0003] Bartomeus, I. , V. Gagic , and R. Bommarco . 2015. Pollinators, pests and soil properties interactively shape oilseed rape yield. Basic and Applied Ecology 16:737–745.

[eap2445-bib-0004] Bartomeus, I. , M. G. Park , J. Gibbs , B. N. Danforth , A. N. Lakso , and R. Winfree . 2013. Biodiversity ensures plant–pollinator phenological synchrony against climate change. Ecology Letters 16:1331–1338. 23968538 10.1111/ele.12170

[eap2445-bib-0005] Bates, D. , M. Mächler , B. Bolker , and S. Walker . 2014. Fitting linear mixed‐effects models using lme4. arXiv preprint arXiv:1406.5823.

[eap2445-bib-0006] Benjamin, F. E. , and R. Winfree . 2014. Lack of pollinators limits fruit production in commercial blueberry (*Vaccinium corymbosum*). Environmental Entomology 43:1574–1583. 25313694 10.1603/EN13314

[eap2445-bib-0007] Bishop, J. , M. P. D. Garratt , and T. D. Breeze . 2020. Yield benefits of additional pollination to faba bean vary with cultivar, scale, yield parameter and experimental method. Scientific Reports 10:2102. 32034193 10.1038/s41598-020-58518-1 PMC7005869

[eap2445-bib-0008] Bishop, J. , H. E. Jones , M. Lukac , and S. G. Potts . 2016. Insect pollination reduces yield loss following heat stress in faba bean (*Vicia faba* L.). Agriculture, Ecosystems & Environment 220:89–96. 10.1016/j.agee.2015.12.007 PMC4767028 26989276

[eap2445-bib-0009] Blaauw, B. R. , and R. Isaacs . 2014. Flower plantings increase wild bee abundance and the pollination services provided to a pollination‐dependent crop. Journal of Applied Ecology 51:890–898.

[eap2445-bib-0010] Blitzer, E. J. , J. Gibbs , M. G. Park , and B. N. Danforth . 2016. Pollination services for apple are dependent on diverse wild bee communities. Agriculture, Ecosystems & Environment 221:1–7.

[eap2445-bib-0011] Breeze, T. D. , et al. 2021. Pollinator monitoring more than pays for itself. Journal of Applied Ecology 58: 44–57.

[eap2445-bib-0012] Brittain, C. , C. Kremen , and A. M. Klein . 2013. Biodiversity buffers pollination from changes in environmental conditions. Global Change Biology 19:540–547. 23504791 10.1111/gcb.12043

[eap2445-bib-0013] Cross, J. , M. Fountain , V. MarkÓ , and C. Nagy . 2015. Arthropod ecosystem services in apple orchards and their economic benefits. Ecological Entomology 40:82–96.

[eap2445-bib-0014] Demestihas, C. , D. Plénet , M. Génard , C. Raynal , and F. Lescourret . 2017. Ecosystem services in orchards. A review. Agronomy for Sustainable Development 37:12.

[eap2445-bib-0015] Dicks, L. V. , et al. 2016. Ten policies for pollinators. Science 354:975–976. 27884996 10.1126/science.aai9226

[eap2445-bib-0016] Eilers, E. J. , C. Kremen , S. Smith Greenleaf , A. K. Garber , and A.‐M. Klein . 2011. Contribution of pollinator‐mediated crops to nutrients in the human food supply. PLOS ONE 6:e21363. 21731717 10.1371/journal.pone.0021363 PMC3120884

[eap2445-bib-0017] FAOStat . 2018. Crops.

[eap2445-bib-0018] Földesi, R. , et al. 2016. Relationships between wild bees, hoverflies and pollination success in apple orchards with different landscape contexts. Agricultural and Forest Entomology 18:68–75.

[eap2445-bib-0019] Garibaldi, L. A. , et al. 2014. From research to action: enhancing crop yield through wild pollinators. Frontiers in Ecology and the Environment 12:439–447.

[eap2445-bib-0020] Garibaldi, L. A. , et al. 2016. Mutually beneficial pollinator diversity and crop yield outcomes in small and large farms. Science 351:388–391. 26798016 10.1126/science.aac7287

[eap2445-bib-0021] Garibaldi, L. A. , N. Pérez‐Méndez , M. P. D. Garratt , B. Gemmill‐Herren , F. E. Miguez , and L. V. Dicks . 2019. Policies for ecological intensification of crop production. Trends in Ecology & Evolution 34:282–286. 30745253 10.1016/j.tree.2019.01.003

[eap2445-bib-0022] Garratt, M. P. D. , et al. 2016. Apple pollination: demand depends on variety and supply depends on pollinator identity. PLOS ONE 11:e0153889. 27152628 10.1371/journal.pone.0153889 PMC4859530

[eap2445-bib-0023] Garratt, M. , et al. 2021. Apple fruit set and quality under contrasting pollination treatments for multiple apple varieties from multiple countries. University of Reading, data set 10.17864/1947.314

[eap2445-bib-0024] Garratt, M. P. D. , J. Bishop , E. Degani , S. G. Potts , R. F. Shaw , A. Shi , and S. Roy . 2018. Insect pollination as an agronomic input: Strategies for oilseed rape production. Journal of Applied Ecology 55:2834–2842.

[eap2445-bib-0025] Garratt, M. P. D. , T. D. Breeze , N. Jenner , C. Polce , J. C. Biesmeijer , and S. G. Potts . 2014. Avoiding a bad apple: Insect pollination enhances fruit quality and economic value. Agriculture, Ecosystems & Environment 184:34–40. 10.1016/j.agee.2013.10.032 PMC3990452 24748698

[eap2445-bib-0026] Garratt, M. P. D. , S. G. Potts , G. Banks , C. Hawes , T. D. Breeze , R. S. O’Connor , and C. Carvell . 2019. Capacity and willingness of farmers and citizen scientists to monitor crop pollinators and pollination services. Global Ecology and Conservation 20:e00781.

[eap2445-bib-0027] Geslin, B. , M. A. Aizen , N. Garcia , A.‐J. Pereira , B. E. Vaissière , and L. A. Garibaldi . 2017. The impact of honey bee colony quality on crop yield and farmers’ profit in apples and pears. Agriculture, Ecosystems & Environment 248:153–161.

[eap2445-bib-0028] Grab, H. , M. G. Branstetter , N. Amon , K. R. Urban‐Mead , M. G. Park , J. Gibbs , E. J. Blitzer , K. Poveda , G. Loeb , and B. N. Danforth . 2019. Agriculturally dominated landscapes reduce bee phylogenetic diversity and pollination services. Science 363:282–284. 30655441 10.1126/science.aat6016

[eap2445-bib-0029] Hothorn, T. , F. Bretz , and P. Westfall . 2008. Simultaneous inference in general parametric models. Biometrical Journal: Journal of Mathematical Methods in Biosciences 50:346–363. 10.1002/bimj.200810425 18481363

[eap2445-bib-0030] Hudewenz, A. , G. Pufal , A.‐L. Bogeholz , and A.‐M. Klein . 2013. Cross‐pollination benefits differ among oilseed rape varieties. Journal of Agricultural Science 152:770–778.

[eap2445-bib-0031] Ingram, J. 2008. Agronomist–farmer knowledge encounters: an analysis of knowledge exchange in the context of best management practices in England. Agriculture and Human Values 25:405–418.

[eap2445-bib-0032] Isaacs, R. , N. Williams , J. Ellis , T. L. Pitts‐Singer , R. Bommarco , and M. Vaughan . 2017. Integrated Crop Pollination: Combining strategies to ensure stable and sustainable yields of pollination‐dependent crops. Basic and Applied Ecology 22:44–60.

[eap2445-bib-0033] Joshi, N. K. , M. Otieno , E. G. Rajotte , S. J. Fleischer , and D. J. Biddinger . 2016. Proximity to woodland and landscape structure drives pollinator visitation in apple orchard ecosystem. Frontiers in Ecology and Evolution 4. 10.3389/fevo.2016.00038

[eap2445-bib-0034] Klatt, B. K. , A. Holzschuh , C. Westphal , Y. Clough , I. Smit , E. Pawelzik , and T. Tscharntke . 2014. Bee pollination improves crop quality, shelf life and commercial value. Proceedings of the Royal Society B: Biological Sciences 281:20132440. 10.1098/rspb.2013.2440 PMC3866401 24307669

[eap2445-bib-0035] Kleijn, D. , R. Bommarco , T. P. M. Fijen , L. A. Garibaldi , S. G. Potts , and W. H. van der Putten . 2019. Ecological intensification: bridging the gap between science and practice. Trends in Ecology & Evolution 34:154–166. 30509848 10.1016/j.tree.2018.11.002

[eap2445-bib-0036] Klein, A. M. , S. D. Hendrix , Y. Clough , A. Scofield , and C. Kremen . 2015. Interacting effects of pollination, water and nutrients on fruit tree performance. Plant Biology 17:201–208. 24731291 10.1111/plb.12180

[eap2445-bib-0037] Klerkx, L. , and J. Jansen . 2010. Building knowledge systems for sustainable agriculture: supporting private advisors to adequately address sustainable farm management in regular service contacts. International Journal of Agricultural Sustainability 8:148–163.

[eap2445-bib-0038] Knapp, J. L. , L. J. Bartlett , and J. L. Osborne . 2017. Re‐evaluating strategies for pollinator‐dependent crops: How useful is parthenocarpy? Journal of Applied Ecology 54:1171–1179. 28781379 10.1111/1365-2664.12813 PMC5516152

[eap2445-bib-0039] Kremen, C. , and A. M. Merenlender . 2018. Landscapes that work for biodiversity and people. Science 362:eaau6020. 30337381 10.1126/science.aau6020

[eap2445-bib-0040] Link, H. 2000. Significance of flower and fruit thinning on fruit quality. Plant Growth Regulation 31:17–26.

[eap2445-bib-0041] Martins, K. T. , A. Gonzalez , and M. J. Lechowicz . 2015. Pollination services are mediated by bee functional diversity and landscape context. Agriculture, Ecosystems & Environment 200:12–20.

[eap2445-bib-0042] Matsumoto, S. 2014. Apple pollination biology for stable and novel fruit production: search system for apple cultivar combination showing incompatibility, semicompatibility, and full‐compatibility based on the S‐RNase allele database. International Journal of Agronomy 2014:9.

[eap2445-bib-0043] Nicholls, C. I. , and M. A. Altieri . 2018. Pathways for the amplification of agroecology. Agroecology and Sustainable Food Systems 42:1170–1193.

[eap2445-bib-0044] Pardo, A. , and P. A. V. Borges . 2020. Worldwide importance of insect pollination in apple orchards: A review. Agriculture, Ecosystems & Environment 293:106839.

[eap2445-bib-0045] Park, M. G. , E. J. Blitzer , J. Gibbs , J. E. Losey , and B. N. Danforth . 2015. Negative effects of pesticides on wild bee communities can be buffered by landscape context. Proceedings of the Royal Society B: Biological Sciences 282:20150299. 10.1098/rspb.2015.0299 PMC4590442 26041355

[eap2445-bib-0046] Pinheiro, J. , D. Bates , S. DebRoy , D. Sarkar , and Team, R.C. 2013. nlme: Linear and nonlinear mixed effects models. R package version 3, 111. https://svn.r‐project.org/R‐packages/trunk/nlme/

[eap2445-bib-0047] Potts, S. G. , et al. 2016. Safeguarding pollinators and their values to human well‐being. Nature 540:220–229. 27894123 10.1038/nature20588

[eap2445-bib-0048] R Core Development Team . 2017. R: A language and environment for statistical computing. R Foundation for Statistical Computing, Vienna, Austria.

[eap2445-bib-0049] Ramírez, F. , and T. L. Davenport . 2013. Apple pollination: A review. Scientia Horticulturae 162:188–203.

[eap2445-bib-0050] Sáez, A. , C. L. Morales , L. Y. Ramos , and M. A. Aizen . 2014. Extremely frequent bee visits increase pollen deposition but reduce drupelet set in raspberry. Journal of Applied Ecology 51:1603–1612.

[eap2445-bib-0051] Samnegård, U. , et al. 2019. Management trade‐offs on ecosystem services in apple orchards across Europe: Direct and indirect effects of organic production. Journal of Applied Ecology 56:802–811.

[eap2445-bib-0052] Smith, M. R. , G. M. Singh , D. Mozaffarian , and S. S. Myers . 2015. Effects of decreases of animal pollinators on human nutrition and global health: a modelling analysis. Lancet 386:1964–1972. 26188748 10.1016/S0140-6736(15)61085-6

[eap2445-bib-0053] Stanley, D. A. , M. P. D. Garratt , J. B. Wickens , V. J. Wickens , S. G. Potts , and N. E. Raine . 2015. Neonicotinoid pesticide exposure impairs crop pollination services provided by bumblebees. Nature 528:548–550. 26580009 10.1038/nature16167 PMC4693958

[eap2445-bib-0054] Stern, R. A. , D. Eisikowitch , and A. Dag . 2001. Sequential introduction of honeybee colonies and doubling their density increases cross‐pollination, fruit‐set and yield in ‘Red Delicious’ apple. Journal of Horticultural Science & Biotechnology 76:17–23.

[eap2445-bib-0055] Sutter, L. , and M. Albrecht . 2016. Synergistic interactions of ecosystem services: florivorous pest control boosts crop yield increase through insect pollination. Proceedings of the Royal Society B: Biological Sciences 283:20152529. 10.1098/rspb.2015.2529 PMC4760166 26865304

[eap2445-bib-0056] Sutter, L. , M. Albrecht , and P. Jeanneret . 2018. Landscape greening and local creation of wildflower strips and hedgerows promote multiple ecosystem services. Journal of Applied Ecology 55:612–620.

[eap2445-bib-0057] Tamburini, G. , R. Bommarco , D. Kleijn , W. H. van der Putten , and L. Marini . 2019. Pollination contribution to crop yield is often context‐dependent: A review of experimental evidence. Agriculture, Ecosystems & Environment 280:16–23.

[eap2445-bib-0058] Vanbergen, A. J. , and the Insect Pollinators Initiative . 2013. Threats to an ecosystem service: pressures on pollinators. Frontiers in Ecology and the Environment 11:251–259.

[eap2445-bib-0059] Webber, S. M. , M. P. D. Garratt , M. Lukac , A. P. Bailey , T. Huxley , and S. G. Potts . 2020. Quantifying crop pollinator‐dependence and pollination deficits: The effects of experimental scale on yield and quality assessments. Agriculture, Ecosystems & Environment 304:107106.

[eap2445-bib-0060] Winfree, R. , J. R. Reilly , I. Bartomeus , D. P. Cariveau , N. M. Williams , and J. Gibbs . 2018. Species turnover promotes the importance of bee diversity for crop pollination at regional scales. Science 359:791–793. 29449491 10.1126/science.aao2117

