# Supplementary material for: Opportunities to reduce pollination deficits and address production shortfalls in an important insect‐pollinated crop
Source: Ecol Appl. 2021 Oct 19;31(8):e02445. doi: 10.1002/eap.2445 (PMC11475340; doi:10.1002/eap.2445)
Supplement: Supplementary file 1 — Appendix S1 [file EAP-31-e02445-s001.pdf]

Supporting Information. Garratt, M.P.D., et al. 2021. Opportunities to reduce pollination deficits and address production shortfalls in an important insect pollinated crop. Ecological Applications.

Appendix S1

Table S1: Details of the data sets involved in the study

Data set	Country	Year	Varieties	Orchard number by variety	Sampling locations per orchard	Open pollination	Pollinators excluded	Supplementary pollination	Early fruit set	Final fruit set	Seed set	Fruit quality	Reference
Ande01	Argentina	2015	Red Delicious	8	10	yes	yes	yes	yes	yes	yes	sugar content	Garratt et al. 2021
Bern01	Australia	2018	Pink Lady	5	4	yes	yes	yes	yes	no	yes	none	Garratt et al. 2021
Blitz01	USA	2013	Various	12	6	yes	no	yes	no	no	yes	width	Blitzer *et al*. 2016
Bore01	Germany	2015	Braeburn, Topaz	30, 3	8	yes	yes	yes	yes	yes	no	width	Samnegård *et al*. 2018
Bosc01	Spain	2015	Golden, Gala, Various	15, 7, 5	5	yes	no	no	yes	yes	yes	width, weight, sugar content, firmness	Samnegård *et al.* 2018
Bosc02	Spain	2015	Golden, Gala, various	15, 7, 5	3	yes	yes	yes	yes	no	no	none	Samnegård *et al.* 2018
Camp01	UK	2013	Amanda, Gilly, Hastings	2, 2, 4	5	yes	yes	yes	yes	yes	no	width	Campbell *et al.* 2017
Garr01	UK	2011	Cox	8	10	yes	yes	yes	yes	yes	yes	width, weight, sugar content, firmness	Garratt *et al.* 2013
Garr02	UK	2012	Cox, Gala	3, 3	30	yes	yes	yes	yes	yes	yes	width, weight, sugar content, firmness	Garratt *et al.* 2014
Garr03	UK	2013	Bramley, Braeburn	3, 2	30	yes	yes	yes	yes	yes	yes	width, weight, sugar content, firmness	Garratt *et al.* 2016
Garr04	UK	2017	Gala	23	3	yes	yes	yes	yes	yes	yes	width, weight, sugar content, firmness	Garratt et al. 2021
Groo01	Netherlands	2013, 2014	Elstar	15	3	yes	yes	yes	no	yes	no	width	De Groot *et al.* 2015
Kirk01	Georgia	2014	Golden delicious, Kekhura, Various, Winter banana	1, 2, 5, 1	1-9	yes	no	yes	no	no	yes	weight	Garratt et al. 2021
Kirk02	Georgia	2015	Kekhura	5	2-7	yes	no	yes	no	no	yes	weight	Garratt et al. 2021
Kova01	Hungary	2012	Relinda	12	5-9	yes	no	no	no	yes	no	width, weight	Földesi *et al*. 2016
Mart01	Canada	2012	McIntosh	20	4	yes	no	yes	yes	no	yes	none	Garratt et al. 2021
Mina01	Spain	2015, 2016	Rego	25	3	yes	no	yes	yes	no	yes	none	Miñarro and García, 2018
Paxk01	Kyrgyzstan	2013	Aport, Kandil, Kirgizski zimni, Livka, Various,	1, 1, 2, 1, 8	1	yes	yes	yes	no	yes	yes	weight	Garratt et al. 2021
Paxk02	Kyrgyzstan	2014	Aport, Golden, Malba, Renet zolotoi, Star crimson, Various	2, 4, 1, 1, 1, 1	1	yes	yes	yes	no	yes	yes	weight	Garratt et al. 2021
Paxk03	Kyrgyzstan	2015	Golden, Montuan, Various	3, 1, 1	1	yes	yes	yes	no	yes	yes	none	Garratt et al. 2021
PaxN01	Northern Ireland	2010	Bramley	25	1	yes	yes	yes	no	yes	no	none	Garratt et al. 2021
Pufa01	Germany	2014	Elstar, Various	1, 25	3	yes	no	yes	yes	yes	yes	width, weight, sugar content	Garratt et al. 2021
Radz01	Germany	2013, 2014, 2015	Golden, Boskoop, Elstar, Idared, Gala	2, 1, 3, 2, 1	1	yes	yes	yes	no	yes	yes	weight	Garratt et al. 2021
Samn01	Sweden	2015	Rubinola, Aroma, Ingrid-Marie, Various	1, 22, 4, 1	3	yes	yes	yes	yes	yes	yes	weight	Samnegård et al. 2019
Vere01	Belgium	2016	Waleffe, Herut, Alkeme, Etoilee, Braibant, Capucin, Rub, Boskoop, Pendu	3, 3, 2, 3, 1, 1, 1, 1, 2	1	yes	no	no	no	no	yes	width, sugar content, firmness	Garratt et al. 2021
Webb01	UK	2014, 2015, 2016	Gala	2	72	yes	yes	yes	yes	yes	yes	width, weight, sugar content, firmness	Garratt et al. 2021

References

Blitzer, E. J., Gibbs, J., Park, M. G., & Danforth, B. N. 2016. Pollination services for apple are dependent on diverse wild bee communities. Agriculture, Ecosystems & Environment, 221, 1-7.

Campbell, A. J., Wilby, A., Sutton, P., & Wäckers, F. L. 2017. Do sown flower strips boost wild pollinator abundance and pollination services in a spring-flowering crop? A case study from UK cider apple orchards. Agriculture, Ecosystems & Environment, 239, 20–29. https://doi.org/10.1016/j.agee.2017.01.005

De Groot, G.A., R. Van Kats, M. Reemer, D. van der Sterren, J.C. Biesmeijer & D. Kleijn, 2015. De bijdrage van (wilde) bestuivers aan de opbrengst van appels en blauwe bessen; Kwantificering van ecosysteemdiensten in Nederland. WENR-report 2636. Wageningen, Wageningen UR, the Netherlands.

Földesi, R., Kovács-Hostyánszki, A., Kőrösi, A., Somay, L, Elek, Z., Markó, V., Sárospataki, M., Bakos, R., Varga, A., Nyisztor, K., Báldi, A. 2016. Relationships between wild bees, hoverflies and pollination success in apple orchards with different landscape contexts. *Agricultural and Forest Entomology* 18, 68-75.

Garratt, M.P.D., Breeze, T.D., Boreux, V., Fountain, M.T., McKerchar, M., Webber, S.M., Coston, D.J., Jenner, N., Dean, R., Westbury, D.B., Biesmeijer, J.C., Potts, S.G., 2016. Apple Pollination: Demand Depends on Variety and Supply Depends on Pollinator Identity. *PLOS ONE* 11, e0153889.

Garratt, M.P.D., Breeze, T.D., Jenner, N., Polce, C., Biesmeijer, J.C., Potts, S.G., 2014. Avoiding a bad apple: Insect pollination enhances fruit quality and economic value. *Agriculture, Ecosystems & Environment* 184, 34-40.

Garratt, M., et al. 2021. Apple fruit set and quality under contrasting pollination treatments for multiple apple varieties from multiple countries. University of Reading Data Archive http://dx.doi.org/10.17864/1947.314

Miñarro, M., García, D. 2018. Complementarity and redundancy in the functional niche of cider apple pollinators. Apidologie 49 (6), 789-802.

Samnegård, U., Alins, G., Boreux, V., Bosch, J., García, D., Happe, A. K., … Hambäck, P. a. 2018. Management trade-offs on ecosystem services in apple orchards across Europe: Direct and indirect effects of organic production. Journal of Applied Ecology, (March), 1–10. doi: 10.1111/1365-2664.13292

Table S2. Linear mixed effect model estimates for the relationship between pollination deficits and pollination service. Orchard, apple variety, study and country were nested random effects

	Value	Std.Error	DF	t-value	p-value
(Intercept)	0.1760803	0.10255146	124	1.716994	0.0885
Yield.service	-0.2635696	0.07750209	124	-3.400806	0.0009

Table S3. Model estimates following post-hoc Tukey tests showing pairwise comparisons for early fruit set under supplementary and pollinator exclusion (closed) treatments for each variety

Variety	Estimate	Std. Error	z value	P value
AmandaSupplementary - AmandaClosed	0.506046	0.129803	3.899	0.0148 *
AromaSupplementary - AromaClosed	0.341779	0.050354	6.788	<0.01 ***
BraeburnSupplementary - BraeburnClosed	0.346387	0.025098	13.801	<0.01 ***
BramleySupplementary - BramleyClosed	0.811475	0.044530	18.223	<0.01 ***
CoxSupplementary - CoxClosed	0.489573	0.039964	12.250	<0.01 ***
GalaSupplementary - GalaClosed	0.652716	0.023080	28.280	<0.01 ***
GillySupplementary - GillyClosed	0.849277	0.145124	5.852	<0.01 ***
GoldenSupplementary - GoldenClosed	1.019266	0.060553	16.833	<0.01 ***
HastingsSupplementary - HastingsClosed	0.731972	0.096749	7.566	<0.01***
Ingrid-MarieSupplementary - Ingrid-MarieClosed	0.015267	0.118493	0.129	1.0000
Pink ladySupplementary - Pink ladyClosed	0.697504	0.091784	7.599	<0.01 ***
RubinolaSupplementary - RubinolaClosed	0.803320	0.236986	3.390	0.0827.
TopazSupplementary - TopazClosed	0.670189	0.167575	3.999	0.0103 *
VariousSupplementary - VariousClosed	0.873552	0.089572	9.752	<0.01 ***

Table S4. Model estimates following post-hoc Tukey tests showing pairwise comparisons for seed number under supplementary and pollinator exclusion (closed) treatments for each variety

Variety	Estimate	Std. Error	z value	P value
AromaSupplimentary - AromaClosed	0.997513	0.096384	10.349	<0.01 ***
BraeburnSupplimentary - BraeburnClosed	1.106226	0.097045	11.399	<0.01 ***
BramleySupplimentary - BramleyClosed	1.236899	0.095462	12.957	<0.01 ***
CoxSupplimentary - CoxClosed	1.101097	0.074586	14.763	<0.01 ***
ElstarSupplimentary - ElstarClosed	1.994938	0.334705	5.960	<0.01 ***
GalaSupplimentary - GalaClosed	1.533900	0.045453	33.747	<0.01 ***
GoldenSupplimentary - GoldenClosed	1.679308	0.358195	4.688	<0.01 **
IdaredSupplimentary - IdaredClosed	1.577909	0.409928	3.849	0.0351 *
RubinolaSupplimentary - RubinolaClosed	1.878280	0.374211	5.019	<0.01 ***
VariousSupplimentary - VariousClosed	2.137089	0.345263	6.190	<0.01 ***

Table S5. Model estimates following post-hoc Tukey tests showing pairwise comparisons for final fruit set under supplementary and pollinator exclusion (closed) treatments for each variety

Variety	Estimate	Std. Error	z value	P value
AmandaSupplementary - AmandaClosed	0.417663	0.092346	4.523	<0.01 ***
AportSupplementary - AportClosed	0.255105	0.168601	1.513	0.9620
AromaSupplementary - AromaClosed	0.115282	0.037789	3.051	0.1181
BraeburnSupplementary - BraeburnClosed	0.165052	0.018473	8.935	<0.01 ***
BramleySupplementary - BramleyClosed	0.430781	0.028609	15.058	<0.01 ***
CoxSupplementary - CoxClosed	0.443310	0.029870	14.841	<0.01 ***
GalaSupplementary - GalaClosed	0.412801	0.016848	24.501	<0.01 ***
GillySupplementary - GillyClosed	0.498743	0.103246	4.831	<0.01 ***
GoldenSupplementary - GoldenClosed	0.410022	0.097342	4.212	<0.01 **
HastingsSupplementary - HastingsClosed	0.460277	0.068831	6.687	<0.01 ***
IdaredSupplementary - IdaredClosed	0.314094	0.216937	1.448	0.9857
Ingrid-MarieSupplementary - Ingrid-MarieClosed	-0.013683	0.088564	-0.154	1.0000
RubinolaSupplementary - RubinolaClosed	0.342176	0.168601	2.030	0.7133
TopazSupplementary - TopazClosed	0.272353	0.084300	3.231	0.0566.
VariousSupplementary - VariousClosed	0.417278	0.085090	4.904	<0.01 ***

Table S6. Model estimates following post-hoc Tukey tests showing pairwise comparisons for apple size under supplementary and pollinator exclusion (closed) treatments for each variety

Variety	Estimate	Std. Error	z value	P value
AmandaSupplementary - AmandaClosed	-0.84645	2.55949	-0.331	1.0000
BraeburnSupplementary - BraeburnClosed	0.58378	0.50623	1.153	0.9988
BramleySupplementary - BramleyClosed	-4.50183	1.29880	-3.466	0.0332 *
CoxSupplementary - CoxClosed	-1.46521	0.99824	-1.468	0.9821
ElstarSupplementary - ElstarClosed	4.74193	1.42318	3.332	0.0508.
GalaSupplementary - GalaClosed	4.25862	0.60891	6.994	<0.01 ***
GillySupplementary - GillyClosed	-2.56019	2.67882	-0.956	0.9999
HastingsSupplementary - HastingsClosed	-2.37572	1.88619	-1.260	0.9966

Table S7. Model estimates following post-hoc Tukey tests showing pairwise comparisons for apple firmness under supplementary and pollinator exclusion (closed) treatments for each variety

Variety	Estimate	Std. Error	z value	P value
BraeburnSupplementary - BraeburnClosed	0.18596	0.28940	0.643	0.99641
BramleySupplementary - BramleyClosed	-0.47755	0.28797	-1.658	0.59712
CoxSupplementary - CoxClosed	-0.40747	0.22193	-1.836	0.46688
GalaSupplementary - GalaClosed	-0.83444	0.13588	-6.141	<0.01 ***

Table S8. Linear mixed effect model estimates for the effects of pollination treatment (Supplementary pollination vs pollinator exclusion) and variety, on apple brix content. Study, orchard, and sampling location were included as nested random effects.

Variety	Estimate	Std.Error	t value
(Intercept)	10.95730	0.53631	20.431
Pollination_treatment	0.19035	0.06949	2.739
VarietyBramley	1.01954	0.69161	1.474
VarietyCox	0.27789	0.60064	0.463
VarietyGala	0.93579	0.55965	1.672
VarietyRed Delicious	0.36118	0.61541	0.587

Table S9. Linear mixed effects model estimates on the effects of variety, seed number and their interaction on apple size. Study, orchard and sampling location within orchard were included as random effects

Fixed effects:	Estimate	Std. Error	t value
(Intercept)	38.266	39.454	1.031
log(Seed.set.mean + 1)	19.787	20.372	0.971
VarietyBoskoop	2.692	8.361	0.322
VarietyBraeburn	27.099	38.767	0.699
VarietyBraibant	5.742	8.546	0.672
VarietyBramley	54.175	38.723	1.399
VarietyCapucin	16.000	9.575	1.671
VarietyCox	26.091	38.394	0.680
VarietyEtoilee	16.838	45.061	0.374
VarietyGala	19.891	38.326	0.519
VarietyGolden	31.667	38.461	0.823
VarietyHerut	-42.817	106.207	-0.403
VarietyPendu	-2.208	6.855	0.322
VarietyRub	12.592	8.361	1.506
VarietyVarious	14.784	38.400	0.385
VarietyWaleffe	2.840	45.061	2.840
log(Seed.set.mean + 1):VarietyBraeburn	-18.887	20.386	-0.926
log(Seed.set.mean + 1):VarietyBramley	-21.709	20.382	-1.065
log(Seed.set.mean + 1):VarietyCox	-19.016	20.380	-0.933
log(Seed.set.mean + 1):VarietyEtoilee	-14.514	24.576	-0.591
log(Seed.set.mean + 1):VarietyGala	-17.443	20.374	-0.856
log(Seed.set.mean + 1):VarietyGolden	-21.527	20.468	-1.052
log(Seed.set.mean + 1):VarietyHerut	30.813	57.520	0.536
log(Seed.set.mean + 1):VarietyVarious	-15.648	20.402	-0.767
log(Seed.set.mean + 1):VarietyWaleffe	4.506	24.576	0.183

Table S10. Linear mixed effects model estimates on the effects of variety, fruit set and their interaction on apple size. Study, orchard and sampling location within orchard were included as random effects

Fixed effects:	Estimate	Std. Error	t value
(Intercept)	0.5752918	0.8412594	0.684
Size.mean	-0.0014826	0.0136203	-0.109
VarietyBraeburn	-0.2990847	0.8511598	-0.351
VarietyBramley	0.3137912	0.8579999	0.366
VarietyCox	0.1353191	0.8526725	0.159
VarietyGala	-0.2242362	0.8437470	-0.266
VarietyGilly	1.2912443	1.1421275	1.131
VarietyGolden	-0.1073290	0.8841164	-0.121
VarietyHastings	-0.2592735	0.8487684	-0.305
VarietyRelinda	-0.4230880	0.8844664	-0.478
VarietyTopaz	1.6059183	1.4257591	1.126
VarietyVarious	-0.2634814	0.8610821	-0.306
Size.mean:VarietyBraeburn	0.0016012	0.0136754	0.117
Size.mean:VarietyBramley	-0.0043563	0.0136917	-0.318
Size.mean:VarietyCox	-0.0035710	0.0137376	-0.260
Size.mean:VarietyGala	0.0024520	0.0136341	0.180
Size.mean:VarietyGilly	-0.0214311	0.0184806	-1.160
Size.mean:VarietyGolden	0.0006588	0.0141214	0.047
Size.mean:VarietyHastings	0.0020943	0.0138863	0.151
Size.mean:VarietyRelinda	0.0053280	0.0142033	0.375
Size.mean:VarietyTopaz	-0.0230160	0.0217729	-1.057
Size.mean:VarietyVarious	0.0036315	0.0138694	0.262
